# Bioequivalence studies of cetirizine tablets using the urine excretion data of healthy Ghanaian male volunteers

**DOI:** 10.1016/j.heliyon.2022.e12665

**Published:** 2023-01-04

**Authors:** Gideon Onuh, Joseph K. Adu, Samuel O. Bekoe, Raphael Johnson, Reimmel K. Adosraku, Samuel Asare-Nkansah

**Affiliations:** a Department of Pharmaceutical Chemistry, Faculty of Pharmacy and Pharmaceutical Sciences, College of Health Sciences, Kwame Nkrumah University of Science and Technology, Kumasi, Ghana; b Department of Pharmaceutics, Faculty of Pharmacy and Pharmaceutical Sciences, College of Health Sciences, Kwame Nkrumah University of Science and Technology, Kumasi, Ghana

**Keywords:** Bioequivalence, Urine excretion data, Cetirizine, HPLC-UV, Pharmacopeia assessment

## Abstract

**Background:**

In the wake of economic challenges, the role of generic medicines has become crucial in meeting the healthcare needs of people. Their use, however, can only be guaranteed if established to be bioequivalent to their corresponding innovator products.

**Aim:**

In this study, we assess the suitability of a generic brand of cetirizine hydrochloride tablet to be used in place of the innovator brand on the Ghanaian market through bioequivalence assessment.

**Method:**

An HPLC bioanalytical method was developed and validated for the detection and quantitation of cetirizine in a urine matrix. This was then used to quantify the amount of cetirizine excreted unchanged in urine samples of 12 healthy male volunteers collected over a 24-h period using a two-way crossover design approach.

**Results:**

Chromatographic separation was successfully achieved with an isocratic elution on a reverse-phase column. The mean retention time for cetirizine was 2.890 ± 0.243 min. The mean cumulative amounts of cetirizine in the reference and test drugs excreted were 5.69 ± 0.98 mg and 5.82 ± 1.96 mg respectively. Other pharmacokinetic parameters including mean relative Areas Under Curve (AUC_0-24_) of 13.32 and 13.05 μg/mL, and peak Concentration (Cmax) of 3.378 and 3.043 μg/mL at the times at which Cmax was observed (Tmax) being 7.25 and 7.42 min were established respectively for the reference and test drugs. The relative bioavailability was determined to be 102.28, making the locally manufactured brand bioequivalent to the innovator brand.

**Conclusion:**

The locally manufactured test Cetirizine drug was found to be bioequivalent with the innovator brand and could serve as a suitable alternative to the latter. Additionally, relevant pharmacokinetic parameters for cetirizine has been established using urinary excretion data.

## 1 Introduction

Global healthcare expenditure has been on a steady rise in recent times [1]. The situation has become even more crucial, with the downturn in economic events in the wake of the COVID-19 pandemic. The outcome has been that, globally, and especially in developing countries, the cost of treatment, and particularly, the cost of medicines has increased significantly [2,3]. In attempts to increase access to essential medicines and contribute to achieving the Sustainable Development Goal (SDG) 3 [4], the policy on generic medicines utilization has been embraced in several countries [5,6]. According to the World Health Organization, generic medicine is defined as a pharmaceutical product that is usually intended to be interchangeable with an innovator product, is manufactured without a license from the innovator company, and is marketed after the expiry date of the patent or other exclusive rights [7]. Generic medicines are generally presumed to be cheaper options than the innovator brands but of similar quality and efficacy as established from bioequivalence investigations [8,9]. As such, in many national healthcare systems, generic medicines are used interchangeably with innovator brands for the treatment of disease conditions [10].

Over the years, the adoption of generic medicine utilization has been fraught with lots of doubts from both healthcare professionals and the public at large but recent reports have shown increasing acceptability and patronage, especially with increased education on the subject and proper regulation by National Medicines Regulatory Agencies (NMRA) [11] coupled with strict adherence to Good Manufacturing Practices (GMP) adopted by generic medicines manufacturers. There is therefore a need to sustain these measures to break all possible barriers to its wide patronage.

The use of antihistamines for relief of symptoms associated with upper respiratory tract infections has surged in the wake of the COVID-19 pandemic. They have been shown to provide beneficial effects among patients infected with the SARS-CoV-2 virus [12,13]. Cetirizine is one of the widely patronized antihistamines due to its less sedative effects. It remains the first-line medication for allergic rhinitis, hay fever, conjunctivitis, eczema, food allergies, and urticaria in most African countries. It is also well tolerated by pregnant and lactating women [14]. Due to its benefits, different generic brands of cetirizine exist on the West African market and are usually cheap and readily available over the counter [15]. A previous study in Albania had shown that generic and innovator brands of cetirizine hydrochloride 10 mg tablets possessed similar physicochemical properties like weight variation, dissolution, friability, disintegration, etc. [16]. Other studies have also demonstrated similar favourable physiochemical properties of cetirizine tablets generally (without emphasis on generic and innovator brands interchangeability) [17,18]. That notwithstanding, the gold standard to demonstrate interchangeability and justify therapeutic equivalence remains in the conduct of bioequivalence assessments [19]. Existing bioanalytical methods have sought to quantify the content of cetirizine in human plasma and breast milk [20,21] but there has not been any report of its application in the conduct of bioequivalence studies. While these procedures are considered far too sophisticated; with some of experimental conditions not easily reproducible in developing settings for routine applications, there is a need to consider cheap alternative approaches. Additionally, challenges exist with respect to ethical considerations in some jurisdictions (for example, developing countries such as Ghana), for the conduct of such assessments with human blood or plasma samples [22]. Additionally, challenges such as lack of robust analytical techniques and access to chemical reference standards, among others, hinder pharmaceutical manufacturers in such environments to conduct bioequivalent studies. Further more, lack of effective regulatory framework regarding generic drug substitution in most developing countries such as Ghana results in such practices by physicians and pharmacists not being backed with informed bioequivalence studies. For these reasons, alternative approaches considered to be equally viable and reliable, for example, using urine samples, could be considered.

We therefore report in the current study the development and validation of a reverse phase HPLC method for bioequivalence study involving locally manufactured cetirizine tablets from human urine samples. The outcome of this investigation would invariably impact policy decisions on the conduct of bioequivalence studies by national regulatory authorities in developing countries such as Ghana and will further provide scientifically needed data to support generic Cetirizine drug interchangeability in the national healthcare system.

## 2 Materials and method

### 2.1 Reagents and materials

Pure cetirizine reference standard (batch No. CTZD005B/15) used in this study was obtained from Pokupharma Company Ltd. (a local pharmaceutical manufacturing company), Kumasi, Ghana. Analar grade of methylene chloride, methanol and glacial acetic acid were purchased from Merck, India. Acetonitrile and trifluoroacetic acid (HPLC grade) used in the study were purchased from Sigma Aldrich. All other reagents including acetone and hydrochloric acid, were of analytical reagent grade. The reference (innovator brand) zyrtec (10 mg) was purchased from Johnson and Johnson Pharmaceutical, USA and test (locally manufactured) histazine (10 mg) was purchased from Ernest Chemist, Accra, Ghana. The drugs were stored at conditions recommended by respective manufacturers.

### 2.2 Instrumentation

Kontron analytical HPLC system with auto-sampler equipped with pump 422 was employed in this study. The HPLC detector was PerkinElmer 785A UV/Vis Detector with Powerchrome 280 software integrator. XBridge C18 with specifications 4.6 × 100 mm in length by the breadth and 3.5 μm pore size stationary phase column was used. The dissolution test was conducted using tablets dissolution test apparatus USP 30 standards. Tablet disintegration test machine by Tab-machines (Mumbai, India) was used for the disintegration test.

### 2.3 Design of the study

#### 2.3.1 Ethical considerations

The Committee on Human Research Publication and Ethics, KNUST, approved the study protocol and issued an ethical clearance certificate (CHRPE/AP/140/20) according to Helsinki Declaration of 1975, as revised in 2007 [23], before the commencement of the study. Signed consent form was obtained from participants after giving them sufficient information on the procedures, risk and benefits of the study.

#### 2.3.2 Selection of subjects

Twelve healthy male volunteers between age 22–35 years with no history of alcohol, drug abuse and non-smokers were selected for the study. The subjects were screened for both physical and medical examination at KNUST hospital and certified to be medically fit for the study according to WHO guidelines [24]. No volunteer was allowed to take any inhibiting agent two weeks before and throughout the study period.

#### 2.3.3 Pharmacopoeial characterization of drug samples

The drug products (reference and test), were subjected to various pharmacopoeial assessments including weight uniformity, melting point, thin layer chromatography (TLC), disintegration, dissolution and content tests. These assessments were performed to ensure that any differences that may be observed amongst the products from the in-vivo studies, are not attributed to differences from sample identity, purity and formulation factors. Weight uniformity, melting point and TLC assessments were all conducted as per pharmacopoeial requirements [25].

#### 2.3.4 Disintegration test

One dosage of the reference and test tablet was placed in 6 tubes each of the basket racks as prescribed by the USP 30. The apparatus was operated in water as immersion fluid at a temperature of 37 ± 2 °C [25].

#### 2.3.5 Dissolution test

The dissolution test was conducted according to the United States Pharmacopeia (USP) dissolution test 3. The medium of dissolution was 900 mL of water at a temperature of 37.0 ± 0.6 °C with a paddle speed of 50 rpm for 30 min. Multiple sampling were performed at specified time intervals and analyzed by UV/Visible spectrometer [25].

#### 2.3.6 HPLC method development and validation

The developed HPLC method involved a chromatographic separation on a guard column (Kinetex 2.6 μm C18 50 × 4.6 mm column Phenomenex) and XBridge C18 column (4.6 × 100 mm, 3.5 μm) and a mobile phase composition of 0.05% Trifluoroacetic Acid (TFA): Acetonitrile (50:50; v/v) in an isocratic mode of elution. Compound of interest (cetirizine) was detected at an optmized wavelength of 231 nm. The developed method was then validated as per International Conference on Harmonization of pharmaceutical products guidelines [26] and the details are as reported below.

#### 2.3.7 Validation of the developed method

##### 2.3.7.1 Specificity

The specificity of the developed method was investigated by analysing a solution of blank urine and comparing the results obtained with a solution of the urine spiked with cetirizine (10 μg/mL). Interferance of cetirizine with components were then assessed, analyzed and recorded.

##### 2.3.7.2 Linearity

A stock solution of 1 mg/mL was accurately prepared. Analyses of diluted solutions of concentrations 1–16 μg/mL were done and chromatograms obtained for the respective concentrations evaluated using regression analysis. All solutions were prepared in the mobile phase.

Limit of Detection (LOD) and Limit of Quantification (LOQ)

The LOD and LOQ were determined from the calibration curve using the equation below.

Equation 1 3.3xϬ/S

and

Equation 2 10xϬ/S

respectively. Where Ϭ is the standard deviation of the response and S is the slope of the calibration curve.

##### 2.3.7.3 Accuracy

The accuracy of the developed method was performed at three concentration levels; 8, 10, and 12 μg/mL of cetirizine solution. Blank urine samples were spiked with an appropriate volume of the stock solution of cetirizine and made up to mark with the mobile phase. The recoveries at each of the concentration terms were then determined.

### 2.4 Precision

The precision determinations carried out included repetability and intermediate precision. The repeatability was carried out from triplicate determinations at three concentration levels of 8, 10 and 12 μg/mL of cetirizine solution in blank urine. Intermediate precision was determined from triplicate determinations at 10 μg/mL on three consecutive days of analyses. In each instance, precision was confirmed by determining the relative standard deviations from the peak areas to be less than 2%.

### 2.5 Robustness

The robustness of the developed method was investigated by varying the flow rate while keeping other parameters constant. The flow rate was varied at 0.9 mL/min 1.1 mL/min and 2.0 mL/min.

### 2.6 In-vivo study

A two way crossover design was used for a single dose (10 mg) of cetirizine in twelve (12) healthy male volunteers. The twelve volunteers were divided into two groups of six subjects. One group was given 10 mg of the reference tablet, while the second group was given 10 mg of the generic brand of cetirizine. After two days of washout period, the administration of the drugs was interchanged between the groups so that at the end of the study, each volunteer would have taken the two study brands of cetirizine. The volunteers were encouraged to fast overnight before administeration of the drug and also abstained from food for 4 h after the drug administration before taking a standard meal. The volunteers were also provided with 200 mL of water at 4 h interval throughout sample collection period [24].

#### 2.6.1 Urine sampling and HPLC analysis

Twelve samples were collected from each volunteer/subject after orally taking 10 mg tablet of cetirizine with strict adherence to all provided protocols. Urine samples were collected at designated time intervals; 0.0, 0.5, 1.0, 1.5, 2.0, 3.0, 7.0, 8.0, 9.0, 13.0, 18.0 and 24.0 h. Two drops of acetic acid were added to each urine sample to help avoid microbial degradation prior to storage. Urine sample containers were sealed with aluminium foil during sample handling to prevent photodegradation of the analyte. Without further pre-treatment, each sample was preconcentrated to 20 mL *in-vacuo* and 1-in-10 dilution performed. The resultant solution was then filtered with a 0.45 μm membrane filter prior to analysis. The concentration of unchanged cetirizine in the urine was determined for each volunteer and also for both reference and test drug products. The amount of cetirizine in each sample and at each sampling time, cumulative amounts and urinary drug excretion profile and rate of urinary excretion profiles of cetirizine (reference and test) were determined [24].

### 2.7 Data Analysis

The comparison of the pharmacokinetic variables was done using GraphPad Prism version 6. The descriptive statistics were conducted at 95% confidence interval limit using IBM SPSS Statistics 21 and a Data Analysis tool pack in Microsoft Excel 2016. A calculated p-value by independent T-test was used to determine the differences between the variables. P-value <0.05 was considered to be statistically significant.

## 3 Results

### 3.1 Pharmacopoeial characterization of drug products

Pharmacopoeial assessment of both generic and test drug were performed. This was to ensure that any variabilities observed between generic and test drugs were not as a result of formulation factors. Results obtained is as indicated in Table 1.

**Table 1:** Pharmacopeia identification test.

	Pure Cetirizine	Reference Tablet	Test Tablet	Aceeptance Criteria
Melting Point (^0^C)	216.5–217	N/A	N/A	208–219
TLC Rf values	0.62	0.60	0.58	N/A
Weight Uniformity	N/A	Passed	Passed	NMT 2 deviations
Dissolution (% dissolved)	N/A	Passed	Passed	NLT 80% (Q)
Disintegration (mins)	N/A	6.20	5.93	NMT 15 min
Assay % Content	99.87	102.7	99.5	99.0–101.0%^a^/95.0–105.0%**

a Content assay reference for pure cetirizine; **Content assay reference for cetirizine 10 mg tablets; N/A - Not Applicable; NMT – Not more than; NLT – Not less than.

### 3.2 Dissolution test

**Fig. 1:**
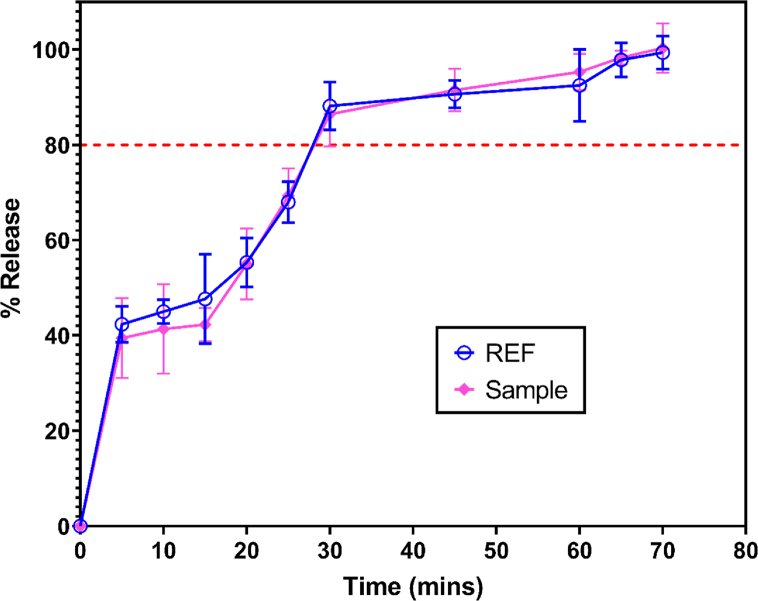
Dissolution profile of Reference and Test Cetirizine Tablets (10 mg). Six (6) replicate determinations at each time point.

The dissolution profile of the reference and test cetirizine tablets passed the United States Pharmacopeia test USP 30. Fig. 1 shows that 80% of the tablets dissolved within 30 min.

### 3.3 Method development and validation

**Fig. 2:**
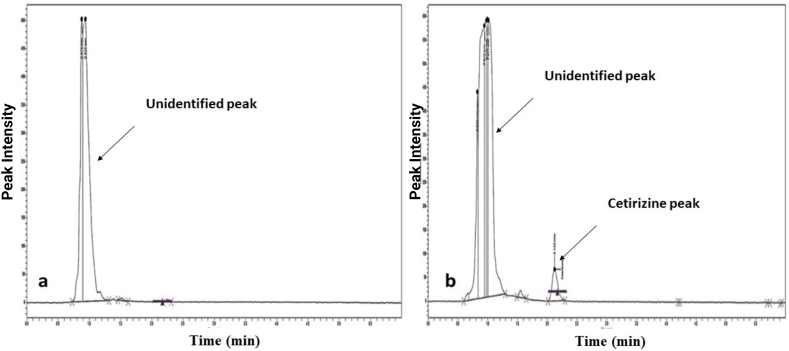
Test for specificity of the method. (a) – Chromatogram of the blank urine, without cetrizine. (b) – Chromatogram of blank urine spiked urine with cetirizine.

The developed method was validated in a urine sample according to the International Conference of Harmonization (ICH) Q2(R1) guidelines. Specificity, linearity, limits of detection (LOD) and quantification (LOQ), accuracy, precision and robustness were all investigated. The method was first investigated for its specificity towards the detection of cetrizine in the presence of the urine. It was observed that in the absence of cetrizine, there was no peak detected at retention time (Rt) ∼2.50 min as shown in Fig. 2a. However, when the urine was spiked with 10 μg/mL of cetrizine, the peak was evident Fig. 2b. The method was therefore specific for the detection of cetirizine. With a high level of correlation between concentration (1–16 μg/mL) and their correspondin peak areas (r^2^ > 0.99, Table 2), the method was also demonstrated to exhibit good linearity for the purposes of quantitation. The LOD and LOQ were also determined to be 0.394 μg/mL and 1.193 μg/mL respectively. For the accuracy of the method, the mean recovery of cetirizine over the concetration range, 8–12 μg/mL was determined to be between 100.5% and 104.0%. The method was also found to be precise (RSD <2.0%) from both repeatability and intermediate precision determinations. The RSD from repatability ranged between 0.44% and 1.82% whiles that of intermediate precision ranged between 0.90% and 1.73% (Table 2). In the robustness test, it was observed that the RSD of determinations at each of the flow rates were less than 2%. However, comparing the responses from each of the flow rates showed significant differences (*p-*value, 0.00032). Thus, varying the flow rate might affect the retention time and the symmetry of peaks obtained for cetirizine beyond the investigated limits. Table 2 summarises the outcomes from the validation investigations. The optmized parameters of cetirizine using the developed HPLC conditions were then successfully applied to the analyses of cetirizine in urine samples.

**Table 2:** HPLC method validation results for Cetirizine.

Parameter	Result	Acceptance criteria
Specificity	Specific	Specific for cetirizine
Linearity		
*Linear dynamic range (μg/mL)*	1–16	
*Regression equation*	y = 18129x + 2075.9	
*Regression cofficient (r*^*2*^*)*	0.9995	>0.99
LOD (μg/mL)	0.394	N/A
LOQ (μg/mL)	1.193	N/A
Accuracy (mean recovery ± SD, %)		
*a. 80% (*8 μg/mL*)*	100.5 ± 3.597	≥85%
*b. 100% (*10 μg/mL*)*	104.0 ± 1.618	
*c. 120% (*12 μg/mL*)*	100.7 ± 2.924	
Precision (% RSD)		
*Repeatability*		
*a.* 8 μg/mL	1.82	RSD not more than 2%
*b.* 10 μg/mL	0.44	
*c.* 12 μg/mL	1.23	
*Intermediate precision (@* 10 μg/mL*)*		
*a. Day 1*	0.90	RSD not more than 2%
*b. Day 2*	1.73	
*c. Day 3*	0.93	
Robustness		
*Δ flow rate*	Robust within working conditions	Robust

### 3.4 Bioequivalence study

**Fig. 3:**
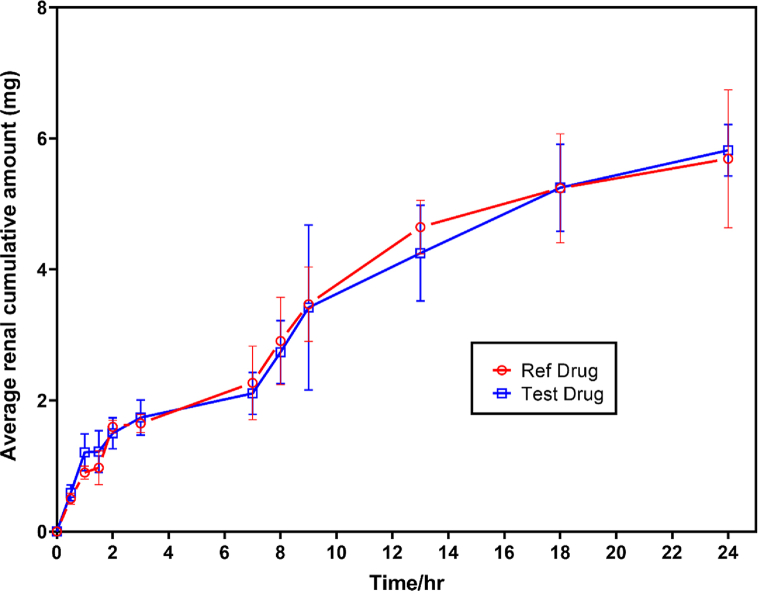
Cumulative renal excretion of cetirizine over 24 h period (test and reference).

**Fig. 4:**
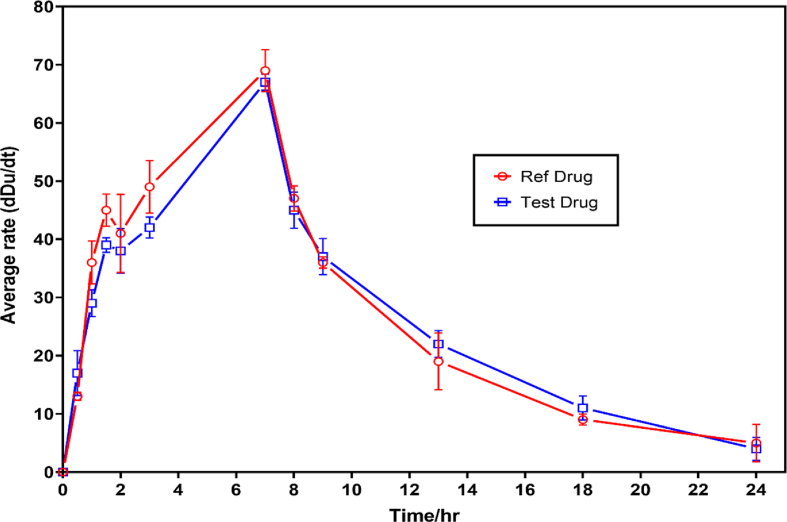
Urinary excretion profile of cetirizine in healthy volunteers (Test and Reference).

Various levels of cetirizine were detected and quantified in urine of the healthy volunteers using the developed and validated method. Cumulative amounts of cetirizine as well as the rate of urinary excretion were then estimated as shown in Figs. 3 and 4, respectively. The relative bioavailability was then determined from data. Relevant pharmacokinetic parameters including the mean relative Area Under Curve from time 0–24 h (AUC_0-24_) of 13.32 and 13.05 μg/mL, peak Concentration (C_max_) of 3.378 and 3.043 μg/mL and time at which Cmax was observed (Tmax) of 7.25 and 7.42 min were established respectively for the reference and test drugs using urine excretion data.

## 4 Discussion

The optimized chromatographic conditions and parameters of validation of the developed method provided results within accepted specifications. The method was thus applied to the analyses of cetirizine in urine of healthy volunteers.

According to the results shown in Table 1, the cetirizine samples used in this study passed the required pharmacopeia tests. The results of the method validation parameters show that the developed method was suitable for the quantification of cetirizine in the urine as compared to the bioanalytical methods reported in the literature. The accuracy, precision, and sensitivity of the developed method were within the acceptable limit for bioanalytical method and was used in the bioequivalence study of cetirizine. Only male volunteers took part in the study. The choice of male volunteers was because of the nature of the sample (urine samples) needed for the study. According to a study conducted by Omolaso et al., [27] studying the effect of Lipton tea, saline load and water load on both male and female urine, it was discovered that the male urine contained fewer biological impurities as compared to the female urine. Moreover, the convenience associated with obtaining urine samples from male volunteers as compared to female volunteers further informed the choice of all male selection. As shown in Figure S1 in the supplementary data, the peak concentration C_max_ and the time at which the peak concentration was observed T_max,_ varied in individual participant who took part in the study. T_max_ of 7.25 ± 0.46 and 7.42 ± 0.69 min were obtained for the reference and test drugs respectively. The average T_max_ of the twelve participants was observed at 7.3 ± 0.6 h. The variation in the pattern of excretion of unchanged cetirizine in the urine of the participants notwithstanding, the homogeneous traits of the subjects could be because of the differences in some physiological factors of the participants such as body temperature, heart rate, serum levels of various stress hormones and immunological functions [28]; Verbeeck et al., 2006). Table 3 illustrates the pharmacokinetics parameters of cetirizine determined using urinary excretion data for the reference and test drugs. The analysis of variance test applied at a 95% confidence interval for varying amounts of cetirizine excreted after the administration of the reference and test drugs showed no significant difference (p < 0.05). This shows that there is no statistical difference in the cumulative mean amount of cetirizine excreted by the participants, despite the slight variation in the amount excreted by the individual participants. Variations in the amount of unchanged drug excreted by the individual participant determined could result from differences in manufacturing processes, choice of excipients and their inherent variations among others and such factors could be the reasons behind the observed variations within the two formulations (Mario et al., 2009). Other factors such as the dissolution rate of the solid dosage form in the gastrointestinal tract (GIT) and the variability in pharmacological effects of a drug on an individual could also justify the slight variation in individual excretion rate of the same dosage tablets [29]. One-way analysis of variance (ANOVA) was conducted at 95% confidence interval for the mean amount of reference and test cetirizine excreted at the various time intervals. A significant difference (*p*-value, 0.012) between the mean concentrations of the unchanged reference and test drugs excreted at the various time intervals was observed. The calculated F-value of 7.603 was higher than the critical F-value of 4.301, which confirms the differences in the mean concentrations of unchanged cetirizine excreted by the twelve subjects at the various time intervals. The determined C_max_ of the locally manufactured test cetirizine and the reference brand of cetirizine was not significantly different statistically (*p*-value, 0.6709). Similar observations were also made on the AUC_0-t_ (*p*-value, 0.6401) and T_max_ (*p*-value, 0.6264) for the test and reference brands of cetirizine. The mean cumulative amounts of cetirizine excreted for the twelve volunteers over the 24 h study period were determined to be 5.69 ± 0.98 mg and 5.82 ± 1.96 mg for reference and test drug products respectively. A relative bioavailability of 102.28 was thus determined making the locally manufactured brand (test drug) bioequivalent to the innovator brand (reference drug).

**Table 3:** Pharmacokinetics parameters of cetirizine in reference and test formulations using urinary excretion data.

Parameter	Reference	Test
*C*_max_ (μg/ml)	3.378 ± 1.621	3.043 ± 0.942 ^ns^
*T*_max_ (h)	7.25 ± 0.46	7.42 ± 0.69 ^ns^
AUC_0–*t*_ (μg ml/h)	13.32 ± 0.96	13.05 ± 0.98 ^ns^

Student *t*-test performed to compare the pharmaceokinetic parameters from both reference and test drugs. ns – not significant.

## 5 Limitations

No internal standard was used for this study due to lack of their access generally in the developing countries. In order to work around this challenge, external standard calibration method was adopted.

## 6 Conclusion

The generic Cetirizine 10 mg tablet as investigated in this study using urine samples from healthy volunteers, possess pharmacokinetic propoerties that guarantees its use in place of the innovator brand on the Ghanaian market. This study also underscores the important role that the use of urine excretory data could be used to study the bioequivalence profiles of generic drugs in places where obtaining data from plasma pose a challenge. Though, it may be considered as a limitation for the study, it also serve as a reliable alternative in such restrictive circumstances.

### Author contribution statement

Gideon Onuh: Performed the experiments; Analyzed and interpreted the data; Contributed reagents, materials, analysis tools or data; Wrote the paper; Joseph K. Adu: Performed the experiments; Analyzed and interpreted the data; Contributed reagents, materials, analysis tools or data; Samuel O. Bekoe; Raphael Johnson: Analyzed and interpreted the data; Wrote the paper; Reimmel K. Adosraku: Conceived and designed the experiments; Contributed reagents, materials, analysis tools or data; Samuel Asare-Nkansah: Conceived and designed the experiments; Wrote the paper.

### Funding statement

Gideon Onuh was supported by 10.13039/501100015212Queen Elizabeth Commonwealth Scholarship, London, United Kingdom [FE-2019-28].

### Data availability statement

Data included in article/supp. Material/referenced in article.

## Declaration of competing interest

The authors declare that they have no known competing financial interests or personal relationships that could have appeared to influence the work reported in this paper.
