# Supplementary material for: Bioequivalence studies of cetirizine tablets using the urine excretion data of healthy Ghanaian male volunteers
Source: Heliyon. 2023 Jan 4;9(1):e12665. doi: 10.1016/j.heliyon.2022.e12665 (PMC9860417; doi:10.1016/j.heliyon.2022.e12665)
Supplement: Multimedia component 1 [file mmc1.docx]

Supplementary Information

**BIOEQUIVALENCE STUDIES OF CETIRIZINE TABLETS USING THE URINE EXCRETION DATA OF HEALTHY GHANAIAN MALE VOLUNTEERS**

Gideon Onuh ^1*^, Joseph K. Adu ^1^, Samuel O. Bekoe ^1^, Raphael Johnson^2^, Reimmel K. Adosraku^1^, Samuel Asare-Nkansah^1*^

^1^ Department of Pharmaceutical Chemistry, Faculty of Pharmacy and Pharmaceutical Sciences, College of Health Sciences, Kwame Nkrumah University of Science and Technology, Kumasi, Ghana

^2^Department of Pharmaceutics, Faculty of Pharmacy and Pharmaceutical Sciences, College of Health Sciences, Kwame Nkrumah University of Science and Technology, Kumasi, Ghana

*Address correspondence to

Gideon Onuh ([gonuh@st.knust.edu.gh](mailto:gonuh@st.knust.edu.gh); +233501336855) and Samuel Asare-Nkansah ([sankansah.pharm@knust.edu.gh](mailto:sankansah.pharm@knust.edu.gh); /+233501571837)

Department of Pharmaceutical Chemistry, Faculty of Pharmacy and Pharmaceutical Sciences, College of Health Sciences, Kwame Nkrumah University of Science and Technology, PMB, Kumasi, Ghana;

**Table S1.** Uniformity of Weight of Reference and Test Cetirizine Tablet

|  | **Reference Sample** | | | **Test Sample** | | |
| --- | --- | --- | --- | --- | --- | --- |
| **Tablet**  **No** | **Weight of**  **Tablet** | **Deviation** | **% Deviation** | **Weight of**  **Tablet** | **Deviation** | **% Deviation** |
| 1 | 0.174 | -0.001 | -0.578 | 0.187 | 0.001 | 0.532 |
| 2 | 0.178 | -0.005 | -2.890 | 0.187 | 0.001 | 0.532 |
| 3 | 0.172 | 0.001 | 0.578 | 0.188 | 0.000 | 0.000 |
| 4 | 0.167 | 0.006 | 3.468 | 0.190 | -0.002 | -1.064 |
| 5 | 0.170 | 0.003 | 1.734 | 0.186 | 0.002 | 1.064 |
| 6 | 0.179 | -0.006 | -3.468 | 0.184 | 0.004 | 2.128 |
| 7 | 0.174 | -0.001 | -0.578 | 0.185 | 0.003 | 1.596 |
| 8 | 0.169 | 0.004 | 2.312 | 0.187 | 0.001 | 0.532 |
| 9 | 0.178 | -0.005 | -2.890 | 0.190 | -0.002 | -1.064 |
| 10 | 0.170 | 0.003 | 1.734 | 0.191 | -0.003 | -1.596 |
| 11 | 0.174 | -0.001 | -0.578 | 0.187 | 0.001 | 0.532 |
| 12 | 0.178 | -0.005 | -2.890 | 0.187 | 0.001 | 0.532 |
| 13 | 0.172 | 0.001 | 0.578 | 0.188 | 0.000 | 0.000 |
| 14 | 0.167 | 0.006 | 3.468 | 0.190 | -0.002 | -1.064 |
| 15 | 0.170 | 0.003 | 1.734 | 0.186 | 0.002 | 1.064 |
| 16 | 0.179 | -0.006 | -3.468 | 0.184 | 0.004 | 2.128 |
| 17 | 0.174 | -0.001 | -0.578 | 0.185 | 0.003 | 1.596 |
| 18 | 0.169 | 0.004 | 2.312 | 0.187 | 0.001 | 0.532 |
| 19 | 0.178 | -0.005 | -2.890 | 0.190 | -0.002 | -1.064 |
| 20 | 0.170 | 0.003 | 1.734 | 0.191 | -0.003 | -1.596 |
| **Total** | **3.462** |  |  | **3.750** |  |  |
| **Mean** | **0.173** |  |  | **0.188** |  |  |

**Table S2.** Assay of Pure Cetirizine

| Abs Min (nm) | E^1^_1_ | Abs Max (nm) | E^1^_1_ | Average E^1^_1_ |
| --- | --- | --- | --- | --- |
| 0.374 | 192.5 | 0.385 | 187 | 189.75 |


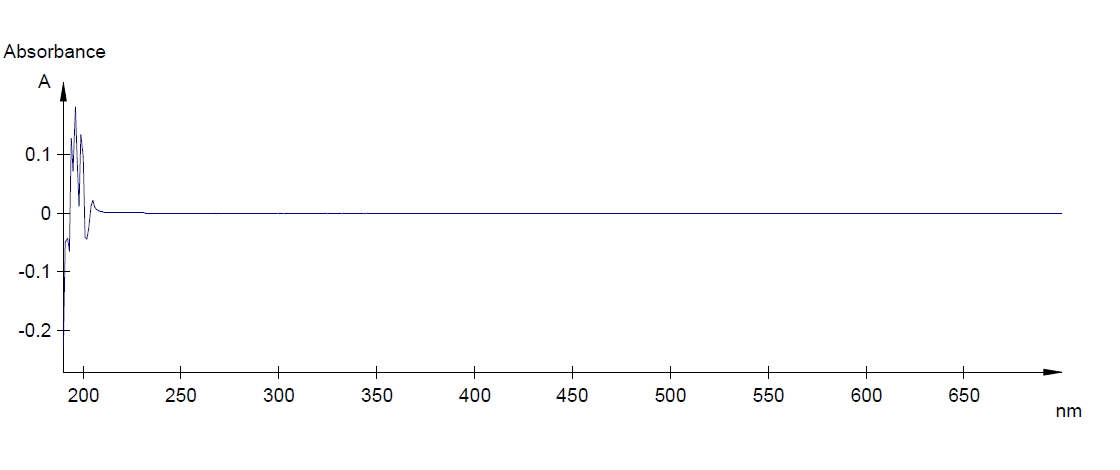


**Figure S1.** UV Spectrum of Blank (solvent)


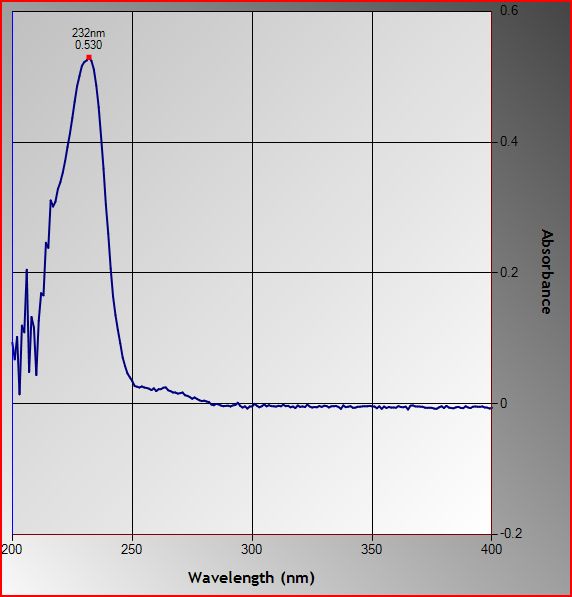


**Figure S2.** UV Spectrum of Pure Cetirizine


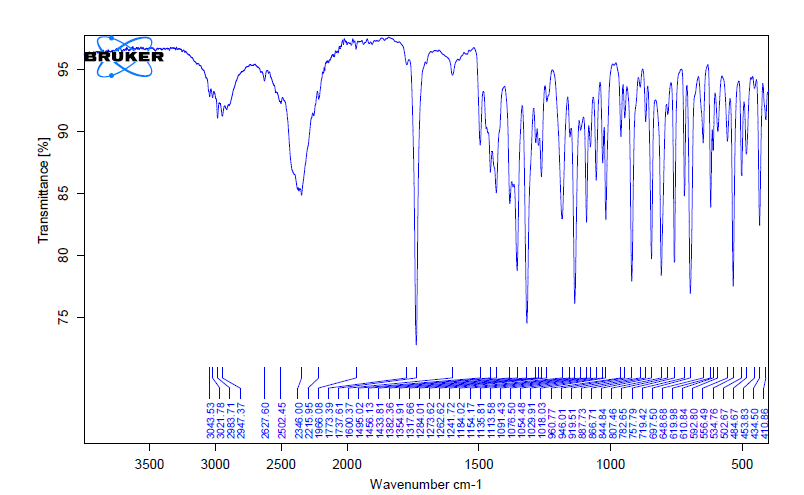


**Figure S3.** IR Spetrum of Pure Cetirizine

**TLC**

10mg in 5mL of water.

Solvent System: Dichloromethane, Methanol, Acectic acid: 70:30:10ml v/v

Solvent Front: 3.7, Distance travelled by pure sample: 1.8, Chlorophenamine maleate 1.5, Reference Sample 1.9 and Test Sample 2.0

$$Rf= \frac{Distance travelled by sample}{Distance travelled by solvent}$$

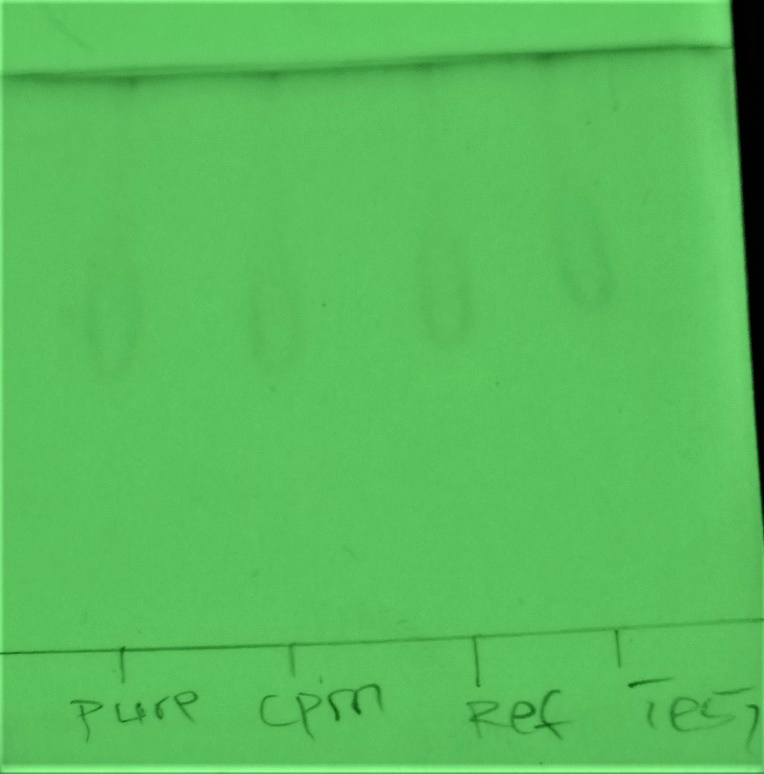


**Figure S4.** TLC Plate

**Table S3.** Dissolution Test

| Time (mins) | Acceptance Criteria (%) | Reference Mean (%) (RSD) | Test Mean (%) (RSD) |
| --- | --- | --- | --- |
| 0 |  | 0.00 (0.00) | 0.00 (0.00) |
| 5 |  | 42.25 (0.79) | 39.47 (0.89) |
| 10 |  | 45.65 (0.45) | 40.86 (0.43) |
| 15 |  | 48.07 (0.34) | 45.06 (0.78) |
| 20 |  | 55.40 (0.54) | 56.17 (0.57) |
| 25 |  | 68.54 (0.45) | 69.56 (0.71) |
| 30 | NLT 80 | 88.00 (0.47) | 86.61 (0.62) |
| 40 |  | 91.81 (0.46) | 92.90 (0.38) |
| 60 |  | 92.85 (0.54) | 95.70 (0.31) |
| 80 |  | 97.44 (0.52) | 98.86 (0.71) |
| 90 |  | 99.90 (0.13) | 99.93 (0.06) |

**Table S4.** Demographics of volunteers

| **Volunteers** | **Group** | **Sex** | **Age** | **Body weight (kg)** |
| --- | --- | --- | --- | --- |
| 1 | xx | M | 26 | 58 |
| 2 | yy | M | 28 | 65 |
| 3 | xx | M | 35 | 71 |
| 4 | yy | M | 24 | 63 |
| 5 | yy | M | 25 | 65 |
| 6 | xx | M | 28 | 62 |
| 7 | xx | M | 28 | 63 |
| 8 | yy | M | 23 | 59 |
| 9 | yy | M | 31 | 69 |
| 10 | yy | M | 28 | 57 |
| 11 | xx | M | 27 | 65 |
| 12 | xx | M | 30 | 61 |

xx: volunteers on test drug before washout period

yy: volunteers on reference drug before washout period
